# Negative news exposure does not affect risk or ambiguity aversion

**DOI:** 10.3389/fpsyg.2025.1485346

**Published:** 2025-06-30

**Authors:** Luis S. Garcia Campos, Karolina M. Lempert

**Affiliations:** Gordon F. Derner School of Psychology, Adelphi University, Garden City, NY, United States

**Keywords:** negative affect, decision making, news, risk avoidance, ambiguity aversion

## Abstract

**Introduction:**

Uncertain decisions can be risky (with known probabilities) or ambiguous (with unknown probabilities). Previous studies have found that negative affect can increase risk aversion and ambiguity aversion, but it is unknown if these effects generalize to more realistic negative stimuli. In real life, negative affect is frequently induced by exposure to news reports. Here, in two pre-registered studies, we examined how watching a negative news video influenced risk and ambiguity aversion.

**Methods:**

Study 1 was conducted online in a sample of university students (*n* = 84), whereas Study 2 was done by a sample on Prolific (*n* = 229). Participants were randomly assigned to one of two groups. The negative news group viewed a news video about a car crash, while the control group watched a news video about train schedules. Then, all participants did a task in which, on each trial, they chose between a certain $5 reward or a gamble option. Half the gambles were risky (e.g., 50% chance of $10; 50% chance of $0), and half were ambiguous, so that the probabilities of the outcomes were not fully known.

**Results:**

Although participants who watched negative news reported a significant increase in negative affect, they did not differ from the neutral news group in their risk or ambiguity preferences.

**Discussion:**

These findings, when considered alongside other similar null findings in the literature, suggest that incidental negative affect might have no effect on decisions under uncertainty, unless the affect is misattributed to the choice itself.

## 1 Introduction

Many of the decisions that humans make involve uncertain outcomes. For example, when deciding whether to participate in a raffle, people do not know ahead of time whether they will win, and the precise probability of winning is often unknown as well. When a person is making a choice that involves uncertainty, one factor that might impact their decision making is their affective state, which can be influenced by their exposure to negative news (McNaughton-cassill, 2001; Thompson et al., 2019). In this study, we examined the effects of watching negative news on decision making under uncertainty.

Uncertain outcomes can be categorized as risky or ambiguous (Ellsberg, 1961). Decisions are risky when the probabilities of the outcomes are known, but the actual outcomes are still dependent on chance. For example, imagine that in a bag of poker chips, there are 30 winning chips and 70 losing chips. If someone pulls a random chip from that bag, they will have a 30% chance of winning and a 70% chance of losing. The exact outcome is unknown, but the probabilities are known. On the other hand, ambiguous decisions are ones in which the probabilities of all outcomes are not known ahead of time. For example, imagine a bag containing 100 chips, in which there are 25 winning ones, 25 losing ones, and 50 chips that are either winning or losing. There is a guaranteed probability of at least 25% of pulling a winning chip, and at least 25% probability of pulling a losing chip, but it is unknown if the remaining chips are mostly losing, mostly winning, or evenly split. People tend to avoid situations that involve uncertain outcomes (Pratt, 1964; Mather et al., 2012). However, people tend to avoid ambiguous situations even more than risky situations (Ellsberg, 1961).

These attitudes toward risk and ambiguity may change when people are in different affective states. For example, mood is an enduring affective state marked primarily by a subjective feeling (Scherer, 2005). It has been proposed that being in a mood leads to the processing of information in a way that is congruent with the mood (Lerner and Keltner, 2001; Slovic et al., 2007; Eldar et al., 2016). If someone is feeling good, then they will generalize that feeling and be more optimistic. If someone is feeling bad, then they are likely to be more pessimistic. It follows that, when someone is in a good mood, they will be more likely to take risks, since they are more likely to believe that the outcome will be in their favor. There is some evidence for this idea. For example, analyses of “big data” show that people buy more lottery tickets when local sports teams have won games, when the weather is sunny, and when the language in Twitter posts is especially positive (Otto et al., 2016; Otto and Eichstaedt, 2018). On the other hand, a negative mood may lead to avoiding risks. When people were feeling negative affect after reading negative statements, they tended to be risk averse (Deldin and Levin, 1986). In another study, people who watched negative movie clips prior to decision making tended to choose more certain options (Hu et al., 2015). Of course, this effect on risk taking may also depend on the type of negative affect elicited; for example, previous research has found that feelings of fear tend to be associated with risk aversion, while feelings of anger are associated with risk seeking (Lerner and Keltner, 2001).

Although there is evidence that affective states influence risk taking, there are also studies showing null results (e.g., Sokol-Hessner et al., 2016). When the probabilities are *known* during risky decision making, it could be that mood has less of an influence on choice, because people do not have to make an initial judgment about how probable an outcome is. In other words, negative affect may increase ambiguity aversion even more so than risk aversion. Some previous research is consistent with this thesis. First, when people are reading a negative story about death, they judge all causes of death as being more probable, showing that they perceive negative events as more likely when they are in a bad mood (Johnson and Tversky, 1983). When people are viewing ambiguous stimuli, such as surprised faces, they are more likely to interpret them as negative if they are anxious (Neta and Brock, 2021). Situations that induce anxiety, an extensive worrisome state (Akiskal, 1998) that has been linked to fear (Reiss, 1987), have shown similar effects on ambiguity intolerance (Eysenck et al., 1991; Richards et al., 2002). Yet another study showed that people become more ambiguity-averse when they are presented with negative stimuli at the time of making a decision (Sambrano et al., 2024). All of these findings can be explained by theories of mood-congruent processing. Because ambiguous probabilities do not present the “full picture” of the chances of an outcome happening (and keeps them hidden from the participant), they are open to interpretation. Participants feeling negative affect may interpret ambiguous situations in a pessimistic manner, which would then make them more likely to avoid gambles with ambiguous outcomes. However, not all research is consistent with this theory, since there have been studies where negative affect-inducing stimuli, such as threat of shock or acute psychological stress, have had no impact on either ambiguity or risk aversion (Sambrano et al., 2022).

Most of the stimuli used to induce negative affect have been administered in the setting of a controlled lab experiment. Some of these stimuli, such as movie clips (Hu et al., 2015), and negative statements (Deldin and Levin, 1986) may not be encountered on a daily basis. In the current study, we examined the effect of induced negative affect on risk and ambiguity aversion using a novel affective manipulation: news reports. According to survey data (Pew Research Center, 2024), 54% of adults in the United States at least sometimes get their news from social media, and two of the most popular platforms for news consumption (YouTube and TikTok) are platforms that specialize in short videos. Negative news has been cited in the literature as one of the most common and consistent causes of negative affect (McNaughton-cassill, 2001; Thompson et al., 2019; Bauldry and Stainback, 2022; Montazeri et al., 2023). Moreover, dynamic stimuli (such as videos) have been found to be more ecologically valid (Schultz and Pilz, 2009; Fox et al., 2009; Sato et al., 2004) and to elicit more affect than static stimuli, such as images (Johnston et al., 2013). Thus, we felt that it was important to examine the extent to which the existing findings generalize to contexts involving dynamic, ecologically valid stimuli that might influence day-to-day decisions under uncertainty.

Although the literature on this topic is mixed, we pre-registered the hypotheses that people who watched a negative news story prior to making decisions would be both more risk avoidant and ambiguity averse compared to people who watched a neutral news story prior to decision making. Of course, it is possible that the negative news exposure would only increase ambiguity, but not risk aversion (Raio et al., 2022). It is also possible that negative news exposure would have no effect on decision making under uncertainty (Sambrano et al., 2022). In Study 1, we investigated this question in a relatively small group of college students. In Study 2, we attempted to replicate our results in a larger, online sample.

## 2 Study 1 materials and methods

### 2.1 Participants

Eighty-one participants completed this online study. Of these participants, seven were excluded for failing attention checks.^1^ One participant was excluded because their responses on the decision-making task were not recorded. Therefore, we analyzed data from a total of 74 participants (mean age = 23.1; SD = 7.07; range = 18–56; 26 Male, 45 Female, 3 non-binary/third gender; Race: 43 White, 6 Black, 8 Asian, 15 Other, 2 Not Reported; Ethnicity: 44 Not Hispanic or Latino, 26 Hispanic or Latino, 4 Not Reported). Thirty-seven participants were randomly assigned to the control group and 37 were assigned to the experimental group.^2^ All participants gave informed consent at the start of the survey. Participants completed the study for course credit (*n* = 39) or on a voluntary basis (*n* = 35). The study was approved by the Institutional Review Board of Adelphi University, and it was pre-registered on the Open Science Framework.^3^

### 2.2 Procedure

**Figure 1:**
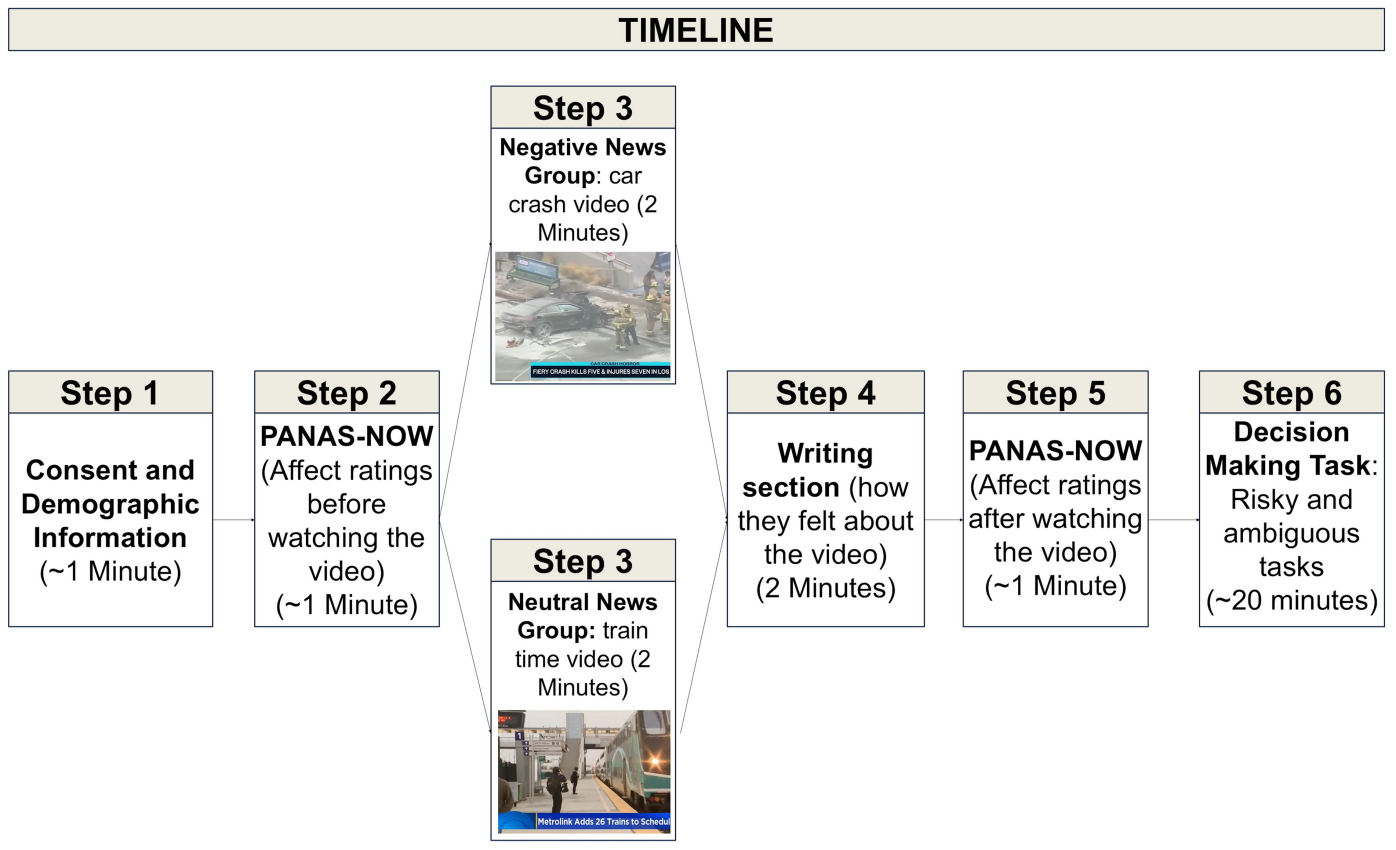
Procedure overview. In this online experiment, participants first gave informed consent and provided demographic information. They then rated their positive and negative affect. After this, half (*n* = 37) of participants watched a negative news video about a car crash, while the other half (*n* = 37) watched a neutral news video about a change in train schedules. Then, both groups wrote about their feelings about the video for 2 min. After making one more set of affect ratings, they did both the risky and ambiguous decision-making tasks, in an order that was randomly assigned.

This was an online study administered using Qualtrics (Provo, UT; see Figure 1 for overview of procedure). After giving informed consent, participants completed a demographic survey. This survey asked about the participants’ age, gender, ethnicity, race, the number of hours they watch news media stories per week, and how much do they think news media influences their day-to-day thoughts (on a 10-point Likert scale).

After this survey, the participants were given the PANAS-NOW questionnaire to assess current levels of positive and negative affect (Watson et al., 1988). Participants rated the extent to which they felt different emotions (e.g., “upset” or “excited”) on a scale from 1 to 5, where 1 means “very slightly or not at all” and 5 means “extremely.” From these data, we calculated a positive and negative affect score, in order to have baseline measurements of positive and negative affect for each participant.

After the PANAS-NOW, participants were instructed that they would next watch a short video about a real news story, and they should pay attention to the video that played automatically. After around 2 min (the approximate length of the news stories), a blue arrow appeared on the screen, letting the participant know that they could proceed to the next screen.^4^

A video of a car crash^5^ was used in order to induce a negative affect state, while a news video about a change in train schedules was used in order to not induce any sort of affective state.^6^ Participants who watched the car crash video were in our experimental (“negative news”) group, while participants who watched the train video were in our control (“neutral news”) group. The videos were chosen based on informal pilot testing, which confirmed that they elicited the affective reactions we expected. We also selected videos that described events occurring far away from the New York area, which is where the data for Study 1 were collected. This was to ensure that participants did not have prior exposure to these videos. After the videos, participants were asked to write about their feelings about the video for 2 min. This writing exercise was meant to reinforce the affective state (Lerner et al., 2004). Then, they did the PANAS-NOW questionnaire again, so that we could measure if their affect changed after watching the video and writing about it.

**Figure 2:**
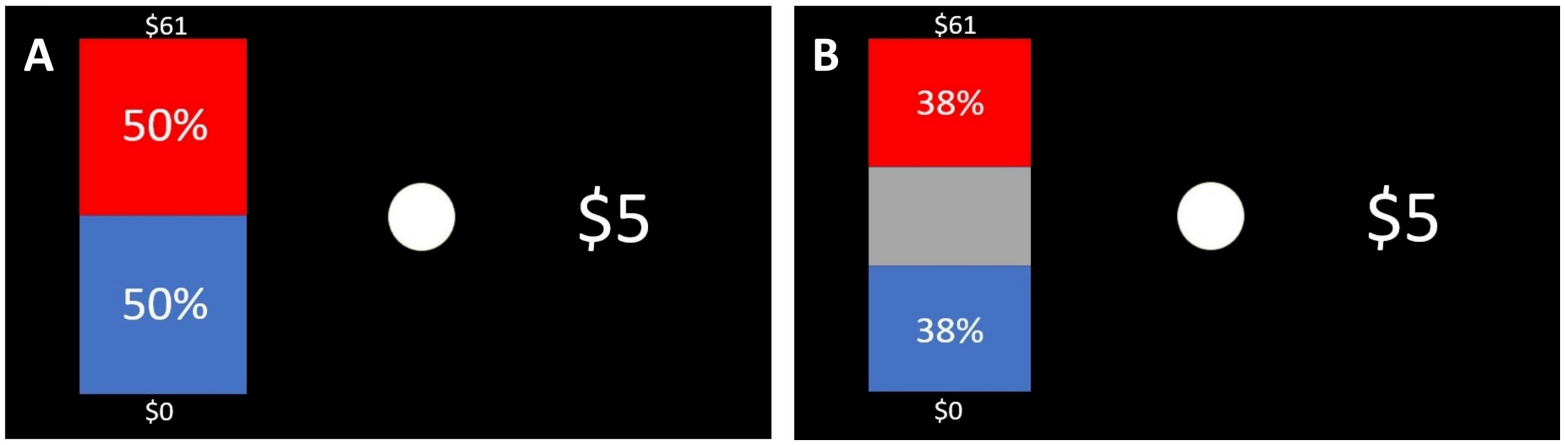
Sample trials from decision-making tasks. For the risk trials **(A)**, the probabilities of getting the larger amount were 50% probability, 25% probability, or 75% probability (randomly intermixed). For ambiguous trials **(B)**, the amount of the graph that was occluded varied – sometimes 5% would be hidden, sometimes 24%, and sometimes 74%. In both cases, one of the options was always $5 for sure. There were 120 trials in total (60 in each task).

After the video, writing exercise, and the second PANAS-NOW questionnaire, participants proceeded to the decision-making tasks. They did both a risky decision-making task and an ambiguous decision-making task (the order was randomized across subjects). These tasks have been validated in other studies as good methods to measure people’s ambiguity and risk preferences (Levy et al., 2010; Raio et al., 2022). Participants saw a series of hypothetical choices. One of the options was always $5 for sure. The other option was a gamble. On risky trials (Figure 2A), the gambling option showed some probability of getting a larger amount in red (e.g., 75% chance of getting $61) and another probability of getting $0 in blue (e.g., 25%). The potential large reward quantities for the task were: ${5, 6, 7, 8, 10, 12, 14, 16, 19, 23, 27, 31, 37, 44, 52, 61, 73, 86, 101, 120}. Each of these quantities was shown three times, paired with each of three different probabilities (50%, 25%, or 75%), for a total of 60 trials. All trials were randomly intermixed. Note that the three trials where the gamble option amount was $5 were considered “attention check” trials, since anyone who is attempting to maximize earnings should not prefer a probabilistic $5 over a certain $5. Any participant who chose to gamble for a potential $5 instead of choosing the certain $5 option three times or more (across both risk and ambiguity tasks) was excluded from analyses.

On ambiguous trials (Figure 2B), the gambling option showed some probability of getting a larger amount (same amounts as in the risky trials) in red and $0 in blue, but there was a gray bar occluding part of the graph, so that the true probabilities of getting the amounts were not known. The proportion of the graph that was occluded varied – sometimes 5% would be hidden, sometimes 24%, and sometimes 74%. However, only the known percentages (red and blue bars) were shown. Here, each of the 20 larger amounts was paired with each of three occlusion levels, for a total of 60 trials in this task as well.

This task was self-paced. Participants did not receive feedback after they made their choices, so the gambles were not actually played out, and participants were not compensated according to their choices.

### 2.3 Analysis

We first ensured that the Negative and Neutral news groups did not differ with respect to age, gender, the number of hours they watched news media per week, or how likely they thought it was that negative media influences their thoughts. We ran an independent samples *t*-test comparing the two groups on age, hours of consumption, and perceptions of media influence on their thoughts. We ran a *χ*-squared test to compare the two groups on gender composition.

Next, we wanted to ensure that the negative news video actually induced negative affect in the experimental group, so we did a repeated-measures ANOVA, with the negative affect score from the PANAS-NOW survey as the dependent variable. The timepoint that the participants completed the PANAS-NOW survey (before vs. after watching the video) was a within-subjects factor, and the group (Negative vs. Neutral) was the between-subjects factor. We expected that negative affect would increase in the negative news group, but not in the neutral news group (i.e., we expected to observe a significant group × timepoint interaction). All ANOVAs described here were followed by post-hoc *t*-tests with Tukey correction.

We also aimed to replicate the consistent finding that people tend to gamble less in ambiguous choices compared to risky choices. To this end, we conducted a paired samples *t*-test^7^ to compare the proportion of choices of the gamble option in the risky trials compared to the ambiguous trials, within-subjects. We expected to see that the proportion of gamble choices would be higher in the risk trials than in the ambiguity trials.

To test if exposure to negative news influenced risk avoidance, we did an independent samples *t*-test, comparing the negative news group with the neutral news group on the proportion of gamble choices in the risky decision-making task. Our expected result was that people in the experimental group would gamble less frequently compared to the control group (i.e., more risk avoidance).

Lastly, we did an independent samples *t*-test to test for an effect of negative news exposure on ambiguity aversion. We compared the negative and neutral news story groups on the proportion of times that they chose to gamble in the ambiguity task minus the proportion of times that they chose to gamble in the risky decision-making task. Subtracting the risky decision-making measure ensures that we are testing for the effects on ambiguity aversion, unconfounded by risk avoidance (Raio et al., 2022). Our expected result was that people in the experimental group would, compared to the control group, show relatively less gambling in the ambiguity task relative to the risk task (i.e., more ambiguity aversion). All statistical tests were performed in Jamovi.

## 3 Study 1 results

There were no significant differences between the groups in age (Negative group: M = 24.1, SD = 8.80; Neutral group: M = 22.2, SD = 4.70; *t*_72_ = −1.17, *p* = 0.246, Cohen’s *d* = −0.272) or gender [χ^2^(2, 74) = 1.50, *p* = 0.471, Cramré’s V = 0.143; Table 1]. There was also no significant difference between groups in either the number of hours that participants watched negative news (Negative group: M = 2.65, SD = 1.18; Neutral group: M = 2.65, SD = 1.32; *t*_72_ < 0.01; *p* > 0.999, Cohen’s *d* = 0.00) or in how much they thought news media influenced their thoughts (Negative group: M = 5.51, SD = 1.92; Neutral group: M = 5.16, SD = 2.22; *t*_72_ = −0.73, *p* = 0.469, Cohen’s *d* = −0.169).

**Table 1:** Characteristics of participants in each group in Study 1 and Study 2.

Characteristic	Study 1: negative news group	Study 1: neutral news group	Study 1 comparison	Study 2: negative news group	Study 2: neutral news group	Study 2 comparison	Between-sample comparison
Gender							
Female *N* (%)	20 (54.05%)	25 (33.78%)	χ^2^_(2, 74)_ = 1.50 *p* = 0.471	61 (26.64%)	61 (26.64%)	χ^2^_(2, 229)_ = 0.339 *p* = 0.844	χ^2^_(6, 303)_ = 4.44 *p* = 0.617
Male *N* (%)	15 (20.27%)	11 (14.86%)		54 (23.58%)	48 (20.96%)		
Non-binary/third gender *N* (%)	2 (2.7%)	1 (1.35%)		3 (1.31%)	2 (0.87%)		
Age							
Age M	24.1	22.2	t_72_ = −1.17 *p* = 0.246	36.65	39.62	t*_227_ =* 1.73 *p* = 0.085	*F*_(3, 299)_ = 31.2 *p* < 0.001
Age SD	8.8	4.7		12.35	13.6		
Age range	[18;56]	[18;35]		[18;69]	[18;70]		
Number of hours of news watched per week							
< 1 h N	7	7	t_72_ < 0.01 *p* > 0.999	12	9	t*_227_ =* 0.206 *p* = 0.837	*F*_(3, 299)_ = 1.20 *p* = 0.310
1–3 h N	15	11		38	30		
3–5 h N	3	9		30	41		
6–10 h N	8	8		22	18		
>10 h N	4	2		16	13		
Self-reported perceived influence of news on thoughts (range: 1–10)							
Perceived influence M	5.51	5.16	t_72_ = −0.73 *p* = 0.469	4.95	5.03	t*_227_ =* 0.267 *p* = 0.790	*F*_(3, 299)_ = 0.67 *p* = 0.572
Perceived influence SD	1.92	2.22		2.21	2.21		

M = Mean. SD = Standard Deviation. For analysis, the number of hours of news watched per week was re-coded as 1–5 (<1 h = 1; 1–3 h = 2; 3–5 h = 3; 6–10 h = 4; > 10 h = 5). Study 1 comparison compares the Neutral News and Negative News groups in Study 1. Study 2 comparison compares the Neutral News and Negative News groups in Study 2. The between-sample comparison column shows test statistics for differences among all four groups from both studies. The only significant difference between studies was in age; Study 2 participants were older on average, since they were drawn from an online community sample.

**Figure 3:**
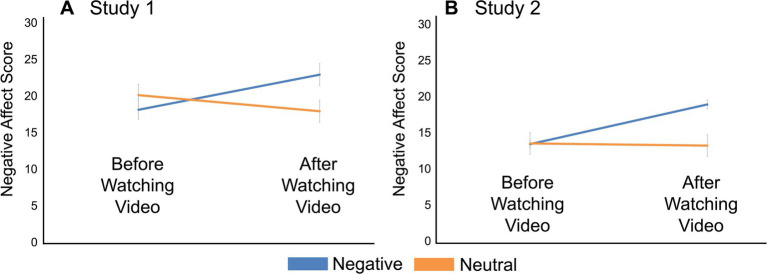
Negative affect results. **(A)** In Study 1, there was a significant increase in negative affect in the negative news group from before to after watching the video (*t*_72_ = −5.02, *p* < 0.001). The neutral group, on the other hand, did not show any change in negative affect from before to after watching the video (*t*_72_ = 2.23, *p* = 0.124). **(B)** In Study 2, negative affect scores of the participants in the negative group increased significantly following the video manipulation (*t*_227_ = −12.04, *p* < 0.001). In the neutral group, negative affect scores did not change significantly (*t*_227_ = 0.23, *p* = 0.996). Error bars indicate standard error of the mean.

As hypothesized, the negative news video significantly increased the negative affect of the participants who watched it. A repeated-measures ANOVA showed that there was no main effect of group (negative vs. neutral), *F*(1,72) = 0.623, *p* = 0.432, partial η^2^ = 0.007, or time point (before vs. after the video), *F*(1,72) = 3.89, *p* = 0.052, partial η^2^ = 0.052. Critically, however, there was a significant interaction between group and timepoint on negative affect, *F*(1, 72) = 26.32, *p* < 0.001, partial η^2^ = 0.038 (Figure 3A). This was driven by a significant increase in negative affect from before (M = 18.1, SD = 8.11) to after watching the video (M = 22.9, SD = 9.17) in the negative news group (*t*_72_ = −5.02, *p_tukey_* < 0.001, Cohen’s *d* = −0.693). There was no significant change in negative affect in the neutral news group (M_before_ = 20.1, SD = 9.10; M_after_ = 17.9, SD = 9.16; *t*_72_ = 2.23, *p_tukey_* = 0.124, Cohen’s *d* = 0.482).

**Figure 4:**
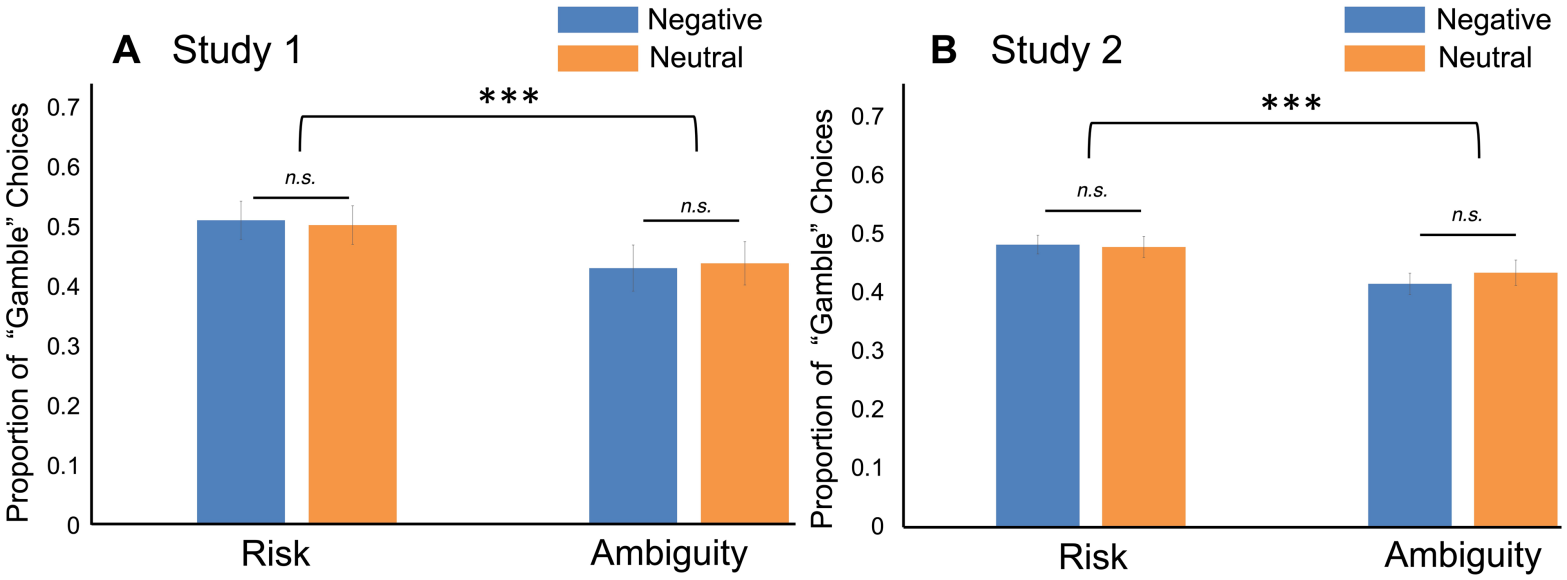
Decision-making results. **(A)** In Study 1, neither risk (*t*_72_ = −0.17; *p* = 0.867) nor ambiguity (t_72_ = 0.5862, *p* = 0.645) preferences were influenced by watching negative news. However, participants were more likely to choose the “gamble” option in the risk trials overall, compared to the ambiguity trials (t_73_ = 4.14, *p* < 0.001). Participants in the Neutral News group selected the gamble option 50.31% of the time on the risk trials, while the Negative News group selected it 51.08% of the time. On the ambiguity trials, the averages were 43.92% (Neutral) and 43.06% (Negative). **(B)** In Study 2, negative affect induced by negative news did not change decision making under risk (*t*_227_ = −0.17, *p* = 0.866) or ambiguity (*t*_227_ = 1.34, *p* = 0.256). In this study as well, participants were more ambiguity averse than risk averse (*t*_228_ = 5.67, *p* < 0.001). Participants in the Neutral News group selected the gamble option 47.72% of the time on the risk trials, while the Negative News group selected it 48.12% of the time. On the ambiguity trials, the averages were 43.27% (Neutral) and 41.43% (Negative). Error bars indicate standard error of the mean. ***p < 0.001. n.s. = not significant.

As predicted, participants were more likely to gamble on the risk trials than the ambiguity trials (*t*_73_ = 4.14, *p* < 0.001, Cohen’s *d* = 0.481; Figure 4A). Contrary to what we originally hypothesized, though, there was no difference in risk avoidance between the negative and neutral groups (Negative group: M = 0.511, SD = 0.195; Neutral group: M = 0.503, SD = 0.197; *t*_72_ = −0.17; *p* = 0.867, Cohen’s *d* = −0.039; Figure 4A). Finally, there were no significant differences between the groups in their decision making under ambiguity (Negative group: M = −0.080, SD = 0.140; Neutral group: M = −0.064, SD = 0.161; *t*_72_ = 0.57, *p* = 0.645, Cohen’s *d* = 0.108; Figure 4A).

Given that we obtained null results when comparing the negative and neutral news groups on risk avoidance and ambiguity aversion, we calculated Bayes factors post-hoc in order to quantify the strength of evidence for the null hypothesis relative to the alternative hypothesis. According to standard cutoffs (Andraszewicz et al., 2015), the Bayes factors indicated that there was moderate evidence for the null hypotheses that risk and ambiguity did not differ between groups (Risk: BF_10_ = 0.243. Ambiguity: BF_10_ = 0.264).

Next, we conducted a few post-hoc exploratory analyses to try to explain our null results. Although the participants in the negative news group showed a significant increase in negative affect *on average*, there were individual differences in the extent to which the news story elicited negative affect. Therefore, it is possible that people who reported more negative affect were more likely to show risk avoidance or ambiguity aversion. However, this was not the case: there was no significant correlation between the change in negative affect and either risk avoidance (*r*_35_ = −0.22, *p* = 0.197) or ambiguity aversion (*r*_35_ = −0.19, *p* = 0.262) in the negative news group.

Another possibility is that the effect may depend on the specific type of negative emotion elicited. Previous research has found, for example, that fear may lead to risk aversion whereas anger may lead to risk seeking (Lerner and Keltner, 2001). Indeed, our manipulation evoked both anger and fear. The average response to the “hostile” item on the PANAS questionnaire (the item most synonymous with “angry”) increased from before to after the manipulation in the negative news group (M_before_ = 1.57; SD = 1.04; M_after_ = 1.95; SD = 1.31; *t*_36_ = −2.34; *p* = 0.025), but not in the neutral news group (M_before_ = 1.49; SD = 0.77; M_after_ = 1.51; SD = 0.87; *t*_36_ = −0.33; *p* = 0.744). There was also a significant increase in the response to the “afraid” item rating in the negative news group (M_before_ = 1.59; SD = 1.04; M_after_ = 2.38; SD = 1.38; *t*_36_ = −3.75; *p* < 0.001), but not the neutral news group (M_before_ = 1.81; SD = 1.22; M_after_ = 1.62; SD = 1.04; *t*_36_ = 1.27; *p* = 0.213). However, the change in hostility from before to after the manipulation was not associated with either risk avoidance, *r*_35_ = −0.017; *p* = 0.920, or ambiguity aversion, *r*_35_ = 0.052; *p* = 0.761, in the negative news group. Similarly, the change in fear was not associated with risk avoidance, *r*_35_ = −0.076; *p* = 0.653, or ambiguity aversion, *r*_35_ = 0.007; *p* = 0.969, in the negative news group. All results remained null when we included the neutral news group in these analyses as well (all *p*’s > 0.05). We also found no significant associations between the post-manipulation hostility and fear ratings and either of our decision-making measures, whether these analyses included all participants or just the negative news group participants (all *p*’s > 0.05).

It is also possible that our null results are due to the negative affect induced by the video wearing off over time. Whereas we did not have access to the exact trial order each participant saw, we randomized whether participants saw the risk or ambiguity block first. We conducted an exploratory ANOVA to examine the effects of group (Negative vs. Neutral) and condition order (Risk first vs. Ambiguity first) on decision making. There were no main effects of group, *F*(1, 70) = 0.01, *p* = 0.924, partial η^2^ = 0.000, or of condition order, *F*(1, 70) = 0.74, *p* = 0.392, partial η^2^ = 0.010, and there was no significant interaction between group and condition order, *F*(1, 70) = 0.10, *p* = 0.750, partial η^2^ = 0.001, on risk avoidance. Similarly, there were no main effects of group, *F*(1, 70) = 0.27, *p* = 0.607, partial η^2^ = 0.004, or condition order, *F*(1, 70) = 0.53, *p* = 0.469, partial η^2^ = 0.007, and there was no significant interaction between group and condition order, *F*(1, 70) = 0.09, *p* = 0.759, partial η^2^ = 0.001, on ambiguity aversion.

## 4 Study 1 discussion

We did not find any significant differences in decision making between the negative and neutral news groups. It is possible that there is no *overall* effect of negative news exposure on decisions under uncertainty, but that the effect depends on individual differences in the affective response. While this did not seem to be the case in Study 1, we did not have sufficient power to properly test this hypothesis, since the negative news group was composed of only 37 participants. Given our small sample size, we also could not rule out the possibility that there *is* an effect of negative news exposure on decision making, but the effect is smaller than we initially predicted. Thus, we conducted Study 2, a replication study with a larger, more representative sample. In Study 2, we expected to replicate the results of Study 1, but in addition, we hypothesized that the change in negative affect within the negative news group would be positively associated with risk avoidance and ambiguity aversion in this group.

## 5 Study 2 materials and methods

### 5.1 Participants

Participants were recruited using the platform Prolific (*n* = 240). We planned to exclude participants who: (1) failed three or more attention check questions, (2) took less than 10 min or more than 60 min to complete the study, or (3) reported not seeing the video. One participant was excluded for failing the attention checks, 9 were excluded because they took more than 1 h to do the study, and one was excluded because they reported that they did not watch the video. Therefore, we analyzed the data of 229 participants (mean age = 38.1; SD = 13.0; range = 18–70; 102 Male, 122 Female, 5 non-binary/third gender; Race: 173 White, 33 Black, 12 Asian, 2 American Indian or Alaska Native, 5 Other, 4 Not Reported; Ethnicity: 201 Not Hispanic or Latino, 24 Hispanic or Latino, 4 Not Reported). One hundred and eighteen participants were randomly assigned to the experimental group and 111 were assigned to the control group. All participants gave informed consent at the start of the survey, and all got paid $6 for doing the study. This study was approved by the Institutional Review Board of Adelphi University. Study 2 was pre-registered on the Open Science Framework.^8^

### 5.2 Procedure

Materials and procedures for Study 2 were identical to those of Study 1 with only two small differences. One was that participants were compensated for their time with money, rather than course credit ($6 for approximately 30 min). Second, participants were asked explicitly at the end of the study if they saw the video, since we wanted to ensure that browser differences (e.g., in auto-play blocking plug-ins) or internet speed differences did not lead some people to not watch the whole video. Finally, we added a setting that would not allow the participant to pause the video while it was playing.

### 5.3 Analysis

The analysis plan for Study 2 was identical to that of Study 1, except that we also pre-registered two correlational analyses. In the negative news group only, we conducted a Pearson correlation between the change in negative affect score (negative affect after watching the video minus negative affect before watching the video) and (1) risk avoidance, and (2) ambiguity aversion.

## 6 Study 2 results

In Study 2, there were no significant differences between the groups in age (Negative group: M = 36.7, SD = 12.3; Neutral group: M = 39.6, SD = 13.6; *t*_227_ = 1.73, *p* = 0.085, Cohen’s *d* = 0.229; Table 1), number of hours that they watched negative news per week (Negative group: M = 2.93, SD = 1.21; Neutral group: M = 2.96, SD = 1.11; *t*_227_ = 0.206, *p* = 0.837, Cohen’s *d* = 0.027), perceived influence of news on their thoughts (Negative group: M = 4.95, SD = 2.21; Neutral group: M = 5.03, SD = 2.21; *t*_227_ = 0.267, *p* = 0.790, Cohen’s *d* = −0.035), or gender [χ^2^(2, 229) = 0.339, *p* = 0.844, Cramré’s V = 0.039].

As we predicted, participants who watched the negative news video reported a significant increase in negative affect. There was a main effect of group, *F*(1, 227) = 13.6, *p* < 0.001, partial η^2^ = 0.044, and of time point, *F*(1, 227) = 63.2, *p* < 0.001, partial η^2^ = 0.038, on negative affect. Most importantly, however, there was a significant interaction between time point and group, *F*(1, 227) = 77.7, *p* < 0.001, partial η^2^ = 0.047 (Figure 3B). Once again, negative affect significantly increased after the manipulation in the negative news group (M_before_ = 13.4, SD = 5.34; M_after_ = 18.9, SD = 6.81; *t*_227_ = −12.04, *p_tukey_* < 0.001, Cohen’s *d* = −0.879), but the same was not true for the neutral news group (M_before_ = 13.5, SD = 6.50; M_after_ = 13.2, SD = 6.51; *t*_227_ = 0.23, *p_tukey_* = 0.996, Cohen’s *d* = 0.094). We also replicated the result that participants gambled less when probabilities were ambiguous rather than known (*t*_228_ = 5.67, *p* < 0.001, Cohen’s *d* = 0.375; Figure 4B).

Just as in Study 1, there was no difference in risk avoidance between the negative and neutral groups (Negative group: M = 0.481, SD = 0.173; Neutral group: M = 0.477, SD = 0.190; *t*_227_ = −0.17, *p* = 0.866, Cohen’s *d* = −0.022; Figure 4B). Moreover, the correlation between the increase in negative affect and risk avoidance was not significant in the negative news group, *r_116_* = 0.08, *p* = 0.390. Just as in Study 1, there was an increase in both fear (the “afraid” PANAS item; M_before_ = 1.28; SD = 0.70; M_after_ = 1.90; SD = 1.09; *t*_117_ = −6.64; *p* < 0.001) and anger (the “hostile” PANAS item; M_before_ = 1.21; SD = 0.58; M_after_ = 1.70; SD = 0.98; *t*_117_ = −5.85; *p* < 0.001) in the negative news group, and no change in either emotion in the neutral news group (fear: M_before_ = 1.27; SD = 0.73; M_after_ = 1.23; SD = 0.74; *t*_110_ = 1.09; *p* = 0.277; anger: M_before_ = 1.28; SD = 0.77; M_after_ = 1.27; SD = 0.71; *t*_110_ = 0.18; *p* = 0.858). However, there was no association between the change in fear and risk avoidance in the negative news group, *r*_116_ = 0.117; *p* = 0.208. We also found no association between the increase in anger and risk avoidance in the negative news group, *r*_116_ = −0.098; *p* = 0.290. These results remained null when including the neutral news group participants in the analysis as well, and when examining associations with only the post-manipulation fear and anger ratings (all *p*’s > 0.05). Therefore, our null result is unlikely to be explained by individual differences in affective reactivity or individual differences in the extent to which the video evoked fear rather than anger.

Lastly, and also in line with the results of Study 1, participants exposed to negative news were not more ambiguity averse than those who watched a neutral news story (Negative group: M = −0.068, SD = 0.149; Neutral group: M = −0.044, SD = 0.150; *t*_227_ = 1.34, *p* = 0.256, Cohen’s *d* = 0.151; Figure 4B). Furthermore, there was no association between the increase in negative affect and ambiguity aversion in the negative news group (*r_116_* = 0.01, *p* = 0.957). There was also no association between the change in fear and ambiguity aversion, *r*_116_ = 0.006; *p* = 0.950, or between the change in hostility and ambiguity aversion, *r*_116_ = −0.005; *p* = 0.955, in the negative news group. These results remained null even when also including the neutral news group participants, and when examining associations with the post-manipulation fear and anger ratings instead of the change scores (all *p*’s > 0.05).

Since we obtained null results in Study 2, we once again calculated Bayes factors post-hoc. These suggested that there was moderate evidence for the null hypotheses that risk and ambiguity aversion did not differ between groups (Risk: BF_10_ = 0.146; Ambiguity: BF_10_ = 0.266). As was the case in Study 1, there were no condition order effects on either risk avoidance or ambiguity aversion.

## 7 General discussion

Here, in two experiments, we investigated the influence of negative affect induced by negative news media on decision making in uncertainty scenarios. In accordance with previous literature (McNaughton-cassill, 2001; Thompson et al., 2019; Bauldry and Stainback, 2022; Montazeri et al., 2023), negative news media increased participants’ negative affect in both studies. In both Study 1 and Study 2, we also replicated the finding that people are less willing to gamble under conditions of ambiguity, compared to when the probabilities of outcomes are known. However, negative affect induced by negative news did not influence either risk avoidance or ambiguity aversion in either study. In Study 2, we found that these null results could not be explained by individual differences in affective reactivity to the negative news story, since there was no correlation between increased negative affect and either risk avoidance or ambiguity aversion.

Some studies have found that people become more risk averse after reading negative statements (Deldin and Levin, 1986) or watching negative movie clips (Hu et al., 2015). However, other studies have shown similar results to ours: no changes in risk preference while under threat of shock (Sambrano et al., 2022) or acute stress (Sokol-Hessner et al., 2016). Regarding ambiguity, previous research has shown that people who scored high in anxiety traits (Neta and Brock, 2021), have experienced anxiety-inducing situations (Eysenck et al., 1991), or have endured many lifetime stressors (Raio et al., 2022) show increased ambiguity aversion. Here we have shown that those results do not generalize to negative affect induced by watching negative news. Thus, our results were more in line with the null findings of Sambrano et al. (2022).

One reason we might not have observed an effect of negative news exposure on decision making is that participants may have attributed their negative affect entirely to the video. Schwarz and Clore (1983) found that, when participants attributed their negative affect to an external stimulus, the impact of the negative affect on choice was diminished. In other words, it could be that negative affect does not incidentally influence choices unless people *misattribute* their negative affect as stemming from the choice. In line with this idea, a recent study (Sambrano et al., 2024) that used a similar task but presented negative stimuli *during* ambiguity choices did find that people were more ambiguity averse when negative stimuli were presented, compared to when neutral stimuli were presented. Under those conditions, people may be more likely to misattribute their negative affect to the choice at hand.

Another possibility is that the effect of negative news on risk taking may depend on the specific emotion evoked by the clip. The appraisal tendency framework, first proposed by Lerner and Keltner (2000), proposes that the impact of an emotion on decision making depends on the appraisal associated with that emotion. Fear leads a person to assess a situation as less certain and controllable, which can manifest as increased risk aversion, while anger, another negative emotion, is associated with a sense of control and certainty, which could elicit more risk taking (Lerner and Keltner, 2001). According to the PANAS scores and to participants’ written descriptions, our manipulation evoked both fear and anger. Therefore, it is possible that those who reacted with relatively more fear became more risk averse, while those who reacted with relatively more anger became more risk seeking, leading the overall effect to be null. While our follow-up analyses with single items on the PANAS did not support this hypothesis, future studies should explore this idea by using stimuli that evoke either fear or anger, but not both.

This study has some limitations. Because this was an online study, we lacked some experimental control, and we cannot be certain that all participants paid attention to the videos they were assigned to watch. Nevertheless, negative affect ratings were consistent with participants having viewed their assigned videos. Another limitation was that the decision-making tasks were hypothetical; there were no real stakes to the choices that participants made. Whereas this is common practice, this meant that there was no incentive for participants to express their true preferences. Therefore, some participants might have rushed through the task. We do not believe that this lack of incentive impacted our results, however. After all, previous studies examining the impact of affect on decision making also used hypothetical choice designs, but still found significant effects (Cassotti et al., 2012; Habib et al., 2015). Next, since we only used one negative news video and only one neutral news video, it is possible that different stimuli (or additional stimuli) would have yielded different results. However, many previous studies of emotion and decision making have also used this single-stimulus approach and have found significant effects (e.g., Lerner et al., 2004). Finally, it is possible that the negative affect induced by the video wore off over time, such that it would only influence the choices that were seen early in the decision-making portion of the experiment. Although we did not have exact trial order information in our study, we did not find any effect of which decision-making block (risk or ambiguity) was seen first by participants. The lack of order effects suggests that negative affect was unlikely to alter choice even when the choices were more proximal to the manipulation.

These limitations notwithstanding, our study showed that, although negative news increased negative affect, this negative affect did not influence participants’ preferences. More research is needed in this field to have a consistent and clear understanding of how emotions can affect decision making. Given how many everyday decisions involve uncertainty, and how often people have to make those decisions when they are feeling negative affect, this is an important research question.

## Data Availability

The datasets presented in this study can be found in online repositories. The names of the repository/repositories and accession number(s) can be found at: Open Science Framework: https://osf.io/tzpvu/.

## Footnotes

^1^This exclusion criterion was not pre-registered in Study 1, so this is a slight deviation from our analysis plan. However, we did pre-register this exclusion criterion for Study 2, so we applied it here for consistency. Results were similar whether these participants were excluded or not.

^2^Note that this sample size is also a slight deviation from our pre-registered plan; we planned to stop when we reached 30 participants per group, but when we received more responses than expected, we felt it was best to include all the data.

^3^ https://osf.io/jwpkd

^4^In the first version of the online survey, the video would play automatically, and when the video finished, the survey would auto-advance to the next section. However, for some participants who had a weak Internet connection, the video started playing late, and did not finish before the next section started. Thus, after the first 36 participants, we changed the survey so that the video would still play automatically, but participants would have to click an arrow to move to the next screen after the whole video had finished.

^5^ https://youtu.be/Y1j2jmkVX6g

^6^ https://youtu.be/WPtOOxZjNsk

^7^Before running *t*-tests, we first confirmed that our measures of risk aversion and ambiguity aversion were normally distributed, according to a Shapiro–Wilk test (Risk: W = 0.978, *p* = 0.222; Ambiguity: W = 0.976, *p* = 0.164). In Study 2, the measures were also normally distributed (Risk: W = 0.998, *p* = 0.060; Ambiguity: W = 0.991, *p* = 0.200).

^8^ https://osf.io/dsnua
